# The Trial of Charles Angus, Esq. for the Murder of Margaret Burns

**Published:** 1809-04

**Authors:** 


### CRITICAL ANALYSIS

OF THE

### RECENT PUBLICATIONS

ON THE

### DIFFERENT BRANCHES OF PHYSIC, SURGERY, AND MEDICAL PHILOSOPHY.

1. *The Trial of Charles Angus, Esq. for the Murder of Margaret Burns.*
2. *A Vindication of the Opinions delivered in Evidence by the Medical Witnesses for the Crown, on a late Trial at Lancaster, for Murder.*
3. *Remarks on a late Publication, entitled, "A Vindication of the Opinions, &c." by* JAMES CARSON, M. D.
4. *An Exposure of some of the false Statements contained in Dr. Carson's Pamphlet, entitled Remarks, &c." in a Letter to that Gentleman, by* JAMES DAWSON, Surgeon.

DEEPLY as we regret the dishonour done to the profession by this unhappy and in many respects ill conducted controversy, we shall not whine over the business by lamenting the necessity we are under of bringing it before the medical public. On the contrary, we shall endeavour, diligently and impartially to explain, and offer our opinion on the whole, as far as it is connected with medicine and medical jurisprudence. No controversy of this kind is unattended with its advantages; and as our readers are chiefly professional, they will not only be interested in the subject, but competent to judge of the merits of each party, as well as of the impartiality of our remarks.

As the trial occupied the court from eight o'clock on Friday morning, till three o'clock on Saturday morning, our readers will readily excuse our noticing any but the medical evidence. The suspicion against the prisoner was, that he had endeavoured to procure a premature delivery, or abortion, by means of an instrument resembling a long trochar, and that he had exhibited, or been privy to the exhibition of certain drugs, which had produced such an effect on the stomach, as in the end proved fatal to the deceased. The symptoms during the life of the deceased were violent vomiting, with intense pain, for nearly 48 hours; after which, these symptoms subsided, and she was discovered by the female servants, removed from the sofa on which they had left her, reclined to one corner of the room, "her face aside the wall, and one foot seemed as if crudled under her." As soon as it was discovered that she was dead, she was removed to the sofa, and placed in a supine posture. It does not appear that any stiffening had taken place, at least not sufficient to prevent the body from being laid straight. This was on Friday morning; about noon noon she was removed to her bed, but no mention is made concerning the stiffening of the body. On Sunday, about noon, the body was examined by Mr. Hay, a surgeon in Liverpool, with his apprentice, in company with Dr. Rutter and Dr. Gerard. The following is his description, encumbered as little as we could contrive with the questions of the council and judge.

" *A.* I did not perceive any thing very particular at first view; on the removal of the body from the bed to the table, on which it was laid, there was a small spot of blood lying on the sheet underneath—not quite so large as my hand.

" We removed the body to the table, and after having drawn the shift that was on her, over her face, we proceeded to the investigation of the body: after having exposed to view the intestines—

" I discovered in the convolutions of the intestines, an olive-coloured fluid.

" Which certainly, I conceived, was not similar to that fluid which we generally find in dead bodies. By the directions of the physicians, I proceeded to examine the stomach: I forgot to mention, that, on opening the body, I discovered a slight inflammation (if you will allow me to use the expression) upon the peritonæal coat, but not sufficient to call a disease.

" It was more upon some of the parts of the intestines, remote from the stomach; and between that and the gut, called the rectum, there were some parts which I could scarcely call inflamed. Bringing the stomach into view, I discovered at the anterior and inferior portion of it (I am speaking now relatively to a person in an erect posture) a preternatural opening; when I discovered it, it was not so large, though very near, as the palm of my hand; perhaps rather larger than a crown-piece, my Lord. I have no doubt, that in drawing down the stomach in the manner I did, that I enlarged the opening. The stomach, which was not distended, but you might call it nearly full, contained a green fluid resembling that which was discovered amongst the intestines, which convinced me that the fluid I discovered below came from the opening in the stomach. It was thought, of course, adviseable to collect some of this fluid, and to submit it to chemical experiment; and having previously provided some wide mouthed bottles, I introduced two into this opening. I suppose from six ounces to half a pound of fluid is what I collected. I then tied the end of the stomach next to the intestines—

*Court.* Near the opening?

" *A.* No, my Lord, much lower down; I suppose four inches lower, and separated the stomach from the body. The stomach then, and the small contents which it had, were put into the water we had made use of to clean the sponges which had soaked up the fluid, extravasated amongst the intestines. There is an intestine that goes from the stomach, called the duodenum, about twelve inches in length, which was certainly much more inflamed than the the other parts of the intestines, and the internal villous coat here was also slightly inflamed. The intestines also contained a great deal of the same fluid that was discovered in the stomach; we included a portion of the intestines between two ligatures; we then opened that portion of the intestine, and caught the fluid in a dish, I believe, or phial, and we set that by, also, for chemical analysis.

*Court*, " Then you have already got three species of fluids, that which you found amongst the intestines, that which you found in the stomach, and that which you found *in* the intestines?

" *A*. Yes, my Lord. I have nothing more to say respecting the stomach, and I will proceed to the womb or uterus—and considering the womb as of a person in an erect posture, as we usually do, its peritonæal coat towards the fundus, or upper part, was slightly inflamed; and on seeing the uterus, which was much larger than natural—we took it out of the body to examine it more minutely; in making an incision the whole length of the side of it, we discovered that it was a healthy uterus that had lately excluded a fœtus from it.

' So far I can form a judgment, that the child must certainly have been nearly full grown. If I had not been particularly called upon to examine this body, and had it come unexpectedly under my eye, I should have said that she had died recently after having expelled a child.

" The vessels, also, going from the uterus to the placenta, for the nourishment of the child, were plainly discoverable.

" *Q*. Then, Sir, am I to take it for your decided opinion, that the child had been very recently expelled from the womb?

*A*. " Yes, Sir; the colour, too, of a very small quantity of fluid, oozing from these vessels, was of a florid colour, which, had she been delivered a few days, would have become much lighter, the colour would have been changed. I have to inform your Lordship, that in a natural state, when a woman is delivered, for a few days afterwards a discharge comes from the uterus, called the lochia. The size of the uterus, which, in its natural state, is not larger than the size of a recent fig, was here, I should conceive, the size of a bullock's heart."

Having giving this very accurate and satisfactory account of the uterus, the council again turn Mr. Hay's attention to the stomach; and here it is but justice to give the whole examination, that the reader may see the manner in which an opinion was *extorted* from him.

" I could wish his Lordship and the Court to understand, that I cannot speak to this question so decisively, as I have done to the others.

" *Q*. But what, in your judgment?

" *A*. In my judgment, I cannot account for that opening from any natural cause.

" *Q*. Can you account for it from any disease with which you are acquainted?

"*A.* No, Sir?

"Q. Was the stomach as strong as a healthy stomach is?

"*A.* The parts above, I mean the cardia, would not yield; but the other parts surrounding this hole, were very pulpy, soft, spongy, and broken down, and I could put my fingers through with much ease, and they would have broke with the slightest touch.

"Q. I believe, in the healthy state, if the stomach is not diseased, or if it has not had something at war with it, it is very tough?

"*A.* It would be difficult to pass your fingers through it, it is a thin bag, but a very dense one.

"Q. Now, Sir, having told me that you know no disease that could produce the effects you have now stated, I am only asking you what, in your judgment, must have been the occasion of them?

"*A.* Why, Sir, having prefaced it by saying, that I cannot account for it by any natural cause, I hope it is not uncharitable to conclude, that some deleterious drug must have been conveyed into the stomach, to produce those appearances.

"Q. Now, Mr. Hay, I will ask you one question; the opening of the stomach you have described; do you think that would occasion death?

"*A.* Most certainly, it was a sufficient cause of death.

"Q. Are you acquainted, Mr. Hay, with the properties of the corrosive sublimate of mercury?

"*A.* Why, Sir, that enquiry would be more properly made when Dr. Bostock is examined."

A cross-examination follows by Mr. Scarlet, in which Mr. Hunter's opinion concerning the digestion of the stomach after death is referred to. But this we shall leave till we come to the publications which appeared after the conclusion of the trial.

A cross-examinatiou also occurs, concerning the probability of fatal hæmorrhage, from a want of contraction of the uterus. This ends only in uncertain conjecture.

Dr. Gerard was next examined, and the following is his account of the probable cause of the corroded stomach,

"I presume the situation of the corroded part is different from what it would have been, if corroded by the gastric juice, being at that part of the stomach which would have been most depending in the erect or sitting positions, particularly when leaning forward in the act of vomiting: it is, of course, that situation in which any thing taken into the stomach would have rested, and acted with most effect during life. I name this, because it had, together with other circumstances, some influence on my opinion. Mr. Hay has mentioned Mr. Hunter's observation, that the stomach of a healthy person is more liable to be affected by that fluid, because in that case it is in a more active state. Miss Burn's having been subject to a violent vomiting for forty-eight hours or more, I could not consider her stomach to be in a healthy state for the secretion of the gastric juice; neither could I consider her death as sudden in the the sense of Mr. Hunter's remarks. It is also to be remarked, that where the gastric juice has been observed, even by Mr. Hunter himself, to have dissolved the stomach, it has always been found to have acted on the contiguous parts, and even to have corroded some of the neighbouring viscera, the diaphragm, which is betwixt the stomach and the lungs, and even the lungs themselves. The quantity of fluid found in the stomach is also of importance, inasmuch as it must have weakened the power of any other liquid, (I need scarcely observe to the Jury) in the same way that water will weaken the strength of spirit by being added to it. I have thought it necessary to make these observations, in order to assign my reason for not considering it to be the effect of the gastric juice, conceiving that to be the only stand that could be made to support a contrary opinion to that which I am about to state, respecting the cause of death. Give me leave to say, that for these reasons, I conclude, and form my opinion upon that conclusion, that the injury sustained could not have been the effect of the gastric juice, but must have arisen from some acrid or poisonous matter taken into the stomach."

The following contains all the important parts of Dr. Bostock's examination.

" Q. What in your judgment had produced that hole ?

*A.* It appeared to me to be produced by some acrid, poisonous substance taken into the stomach ?

" Q. Give me leave to ask you, Dr. Bostock, whether corrosive sublimate of mercury, taken in solution, would have produced this effect ?

" *A.* That is a question I cannot exactly answer, for I have had no experience of the effect of its being taken, where the stomach has been afterwards dissected, excepting some cases which are not completely analogous—the nature of the stomach in these cases being somewhat different. I am, however, of opinion, that poison would produce such an effect,"

Dr. Bostock, after this, relates instances in which he had poisoned dogs with corrosive sublimate; yet, after death, could find no traces of that substance in the contents of the stomach.

On the part of the defence, the only medical evidence is Dr. Carson's, which we shall give as fully as the nature of our limits will admit. After stating, that in his opinion the perforation of the stomach must have taken place after death, he observes,

" I cannot conceive any poison of so acrimonious nature taken into the stomach, as to produce a hole of the magnitude that this was, without at the same time producing other destructive appearances throughout the whole surface of the stomach; for, from the agitation of the body necessarily excited under violent pain of the stomach, and from the action of vomiting, this poisonous substance must have been tossed from one part of the stomach to another.——Farther, upon the supposition that this hole had been occasioned by acrimonious poison, it must have been occasioned occasioned when that poison was in the greatest quantity, and greatest concentration on the stomach; therefore, that hole, as the poison was all washed out of the stomach at the time of death, and none was to be found there, nor in the intestines, must have been produced some time before death, if occasioned by an acrimonious substance, and in that case the greater part of the fluid that had been taken into the stomach must have passed through this hole into the general cavity of the belly.—Besides, my Lord, in this case, those symptoms do not appear which usually accompany such a horrible poison as this must have been.

"From all the histories I have read of mineral poisons, I am led to believe that the most violent convulsions have always preceded death, accompanied with great anguish and the most horrible pains, such as have been by no means described in this case. The three great constituents, then, which form the proof of poison, namely, the existence of poison in the stomach itself, which is the strongest; the appearances suitable to such poison, upon dissection; and the symptoms which accompany the action of it, previous to death, are not found. It has generally been supposed by cautious men, that a plain proof of poison cannot be maintained, and plainly declared, unless in a case where these circumstances are found combined. But a hole in the stomach after death is by no means an uncommon appearance. We are informed by Morgagni, Mr. John Hunter, and other eminent authorities, that there are various instances of a hole being found in the stomach, when no previous disease could have been supposed to exist, or any other acrimonious poison taken into the stomach.

"There are three particular cases, mentioned by Mr. Hunter, which I will briefly state. The first was the case of a man who had been killed by a stroke on the head from a poker, and had died instantly. He had eaten a plentiful supper of beer, bread, cheese, and animal food. Now in this case, the influence of the gastric juice, is out of the question. Mr. John Hunter's inference is wrong, though the fact stands good; the fact is, the stomach was corroded, and the contents of it were found, upon dissecting the body, to have passed into the cavity of the belly, in contact with the spleen, liver, &c. The next case in which he observed this appearance, was in the stomach of a man, whose skull had been fractured by a brick bat. Now the circumstances were more remarkable here, for not only was the stomach corroded, but the adjacent side of the spleen was consumed, the diaphragm was even perforated, and the contents of the stomach were found in the cavity of the thorax, in contact with the lungs. The third case, which he particularly mentions, was that of a soldier who had been executed. Now, Sir, it appears to me, that this appearance is to be accounted for in the following way. From the experiments of Sir John Pringle, which were afterwards confirmed by Dr. M'Bride, we know that water, at the temperature of 90 degrees, especially if that portion of common salt which we usually usually take with our food be mixed with it, will dissolve animal substances in fourteen hours. Heat, moisture, and confined air, from the experiments of these men, are the great promoters of the solution of animal substances. Now, my Lord, in the ordinary cases of death, the vital principle is not destroyed till the heat of the body is reduced to a low temperature, nearly to that of the surrounding air; but in cases of sudden death, the vital principle is destroyed when the heat of the body is still at the standard of 96 degrees. As the human body is a slow conductor of heat in the stomach, there may have been such a degree of heat, combined with liquid aad confined air, as to dissolve the parts in contact with the fluid."

The following is Dr. Carson's deposition concerning the Uterus.

"Notwithstanding the confidence with which all my medical friends agree upon this subject, there are certain circumstances which render it at least doubtful in my mind—as, for instance, the very great dilated state of the cavity of the uterus; for it is well known that the reason why women do not flood to death, at the time of the separation of the placenta, is the contraction of the womb. In a very short time after delivery, the womb contracts so as almost to abolish its cavity. The womb indeed is larger after delivery, than in the unimpregnated state, but that arises from the thickness of the walls of the womb, not from the extent of the cavity itself; for in those cases in which the cavity is not contracted, there is always a great flooding, and it appears to me that if this womb had lately parted with a placenta, the mother must have either flooded to death, or the womb must have been gorged with coagulated blood. I think, that the most probable case, independent of pregnancy, is a dropsy of the hydatids, a common complaint, and of which Astruc gives many instances. These hydatids are attached by pedunculi to the internal surface of the womb, and when by an action being excited in the womb, similar to parturition, these hydatids are expelled, the mouth of the womb is dilated.

"Q. Now in the case of a dropsy of the hydatid kind, as you have stated, would the womb be left in the enlarged state, without contraction?

"*A.* Though it should not contract very much, the vessels nourishing the hydatids may be supposed so much smaller than those nourishing a fœtus, that, in a state of undue contraction, such a flooding may not take place upon the expulsion of the hydatids.

"Q. Well, then, I understand that, in your judgment, the distended cavity of the womb, unaccompanied either by coagulated blood within, or the circumstance of a flooding, can only be accounted for in the way you have described—namely, a dropsy of the hydatids?

"*A.* I cannot say it is the only way; but if neither of the two circumstances I have stated took place, it is physically impossible sible that that womb could have parted with a placenta. If the woman had not died of a flooding, or of no coagulated blood, compressing and plugging up the vessels of the womb, was found on examination of the uterus, then, I say, it is physically impossible that it could have parted with a placenta.

"Q. Now, Sir, do you agree with Mr. Hay in his statement that the woman did not die of a flooding?—*A.* I certainly do."

The next Pamphlet is announced under the joint names of Drs. Gerard, Rutter, and Bostock, and Mr. Hay. By an Introduction it appears to have been written in consequence of an intention, expressed in writing, by Dr. Carson, that "he is preparing a Statement of the whole Case to be submitted to the Colleges of Physicians and Surgeons at London and Edinburgh, and for publication." These Gentlemen therefore, thought it right to begin, first, with an accurate statement of the medical evidence. This they divide into three parts: First, the narrative of the circumstances attending the examination of the body, and a statement of their opinions on the appearances discovered, supported by practitioners of the highest characters in London and Liverpool. Secondly, An examination of Dr. Carson's evidence; and, Lastly, General observations on the subject, and on medical evidence.

At the end of the Report is the following paragraph.

"From the time when we drew up this Report, on the 30th of March, until the day of trial on the 2d of September, we had not the most distant conceptiou that any person could be produced, who, after having seen the Uterus, would attempt to explain the state in which it was found, on any other grounds than those of pregnancy, and subsequent delivery of a child. The Uterus, as well as the stomach, both of which had been taken from the body of the deceased, were shewn, without the least reserve, to every medical gentleman who had a wish to see them; and, indeed, we were desirous that they should be inspected and examined by others of the profession besides ourselves; and not one individual, who saw the Uterus, ever expressed, as far as we know, the slightest doubt upon the subject of Miss Burns' pregnancy.—Dr. Carson saw the Uterus amongst others, and the stomach also; but at that time, although he knew our opinions on the subject, he expressed no doubt about the pregnancy; nor was one word then said by him about hydatids. If any proof could be established by inspection of the Uterus, that its growth and enlargement had depended upon hydatids, that proof must have been the strongest whilst it was comparatively recent: yet he gave no hint of this when he saw the Uterus; nor did such a thought enter into the mind of any other man who saw it; and it was seen by a considerable number of professional gentlemen."

After this, the reasons are stated for the opinions formed concerning the appearances in the stomach and uterus. Of these, we shall at present only remark, that subsequent to the trial, during which they learned, for the first time, that doubts had been entertained tained of the pregnancy of the deceased, they hed examined the ovaria, and discovered a *corpus luteum*. Mr. Hay brought the uterus and ovaria to London, where he exhibited them to the following gentlemen, Drs. Denman and Haighton, Messrs Cline, A. Cooper, Abernethy, and C. M. Clarke. The unreserved opinion of each, given severally and distinctly, is, that they know of no means of accounting for the appearances but by an advanced state of pregnancy in the deceased. Next are added the testimony of seventeen gentlemen, surgeons, accoucheurs, or physicians in Liverpool, all to the same conclusion.

Several remarks follow, on the evidence given by Dr. Carson; but these we shall reserve till we offer our own opinions. From the general observations which conclude the pamphlet, we extract the following, as sufficient for every purpose with a medical reader.

"In order to place the matter in a proper light, let us suppose that another instance of death had occurred under circumstances precisely similar to that of Miss Burns; in which all the subsequent proceedings were similar; and that another medical practitioner, in habits of frequent intercourse with one of those who had examined the body, had formed a different opinion, with respect to the cause of the appearances observed. Now, the question is, what ought to be his conduct on such an occasion? He may be considered as a calm and indifferent spectator, who may have had time to form his opinions, without having his mind disturbed by fears and apprehensions, of falling into error, of being deceived or misled by appearances, or of bringing the life of, perhaps, an innocent person into danger. Ought he not, before the day of trial, to go to his brethren, who have been concerned in the investigation, and to represent to them his doubts, with respect to the correctness of their conclusions, and thus to give them an opportunity to rectify their opinions before it be too late? Is not this the conduct which an upright and honourable man would pursue? Would not such a man take all possible pains to endeavour to convince his brethren, that they were under a mistake, and to persuade them to reconsider their opinions carefully, so as not to incur the dreadful responsibility of precipitating, by a hasty and unguarded judgment, a fellow creature to destruction? One thing, at least, is certain, that such conduct could never, under any possible circumstances, be wrong, or be productive of bad consequences to any party.

"Now, what was Dr. Carson's conduct on the late occasion? Did he act in this manner? Did he communicate his doubts to his brethren? Did he at any time give a hint, even the slightest hint, to any one of us, that he entertained any doubts at all on the subject? During the whole of the five months, which elapsed from the death of Miss Burns, to the trial of Mr. Angus, not one of us even had a suspicion, that his opinions were different from ours. Even Mr. Hay, who was on terms of intimacy and friendship with him, and was occasionally at his house, never heard from him, the least intimation of the kind. Nay, when Mr. Hay met him on the walls walls of Lancaster Castle, two days only previous to the trial; *Dr. Carson told him, that he did not know why he had been summoned up to Lancaster.* This silence and reserve with respect to his opinions, for so long a period, and this declaration, were enough to lull asleep the most vigilant suspicion, even if we had suspected that he had differed from us.

"After the declaration which he had made to Mr. Hay, that he did not know why he had been summoned to Lancaster, great, indeed, was Mr. Hay's astonishment, when, on the day of trial, he saw him in court seated by the side of Dr. Campbell, of Kendal,* immediately behind the prisoner's counsel; and greater still, when he observed him and his coadjutor dictating questions to the prisoner's counsel, during the cross examination of the medical witnesses for the crown. Not that we conceive there was any thing improper in their conduct in assisting counsel to investigate the truth; but their appearance in that particular situation, was a decisive proof of the part which they had taken; and it is also a strongly presumptive proof that Dr. Carson, notwithstanding his declaration to Mr. Hay, did know why he had been summoned to Lancaster. But there is no necessity to have recourse to presumptive proofs of this fact."

Dr. Carson, in his remarks on the above publication, uses a severity, and sometimes a flippancy of language, which are unbecoming his subject. If he was provoked into the first, no excuse can be made for the last.

"If the destruction, (says D. C.) discovered in this stomach had been occasioned by some deleterious drug, this effect must have been produced when the poison existed in the greatest quantity and concentration in the stomach. At the time of death it had been washed away, so that not the least quantity of it remained, therefore this aperture would have existed some time before death; and the liquids that had been taken into the stomach immediately before death, would have passed through this hole into the general cavity of the belly. But only a very small quantity was found in the convolutions of the intestines, while the stomach itself was full. The authors of the "Vindication" have endeavoured to explain away this objection, by supposing that the hole might not have actually taken place till after death, although the injury to the substance had. But if any part of the stomach had been, some time before death, in so tender a state as easily to admit a passage to the fingers, the action of vomiting, which, when violent, as is described by them to have been the case, in this instance, sometimes

"* We are surprised at the part which this gentleman took in the affair. He never saw the appearances; nor, as far as we have been able to learn, did he write to one of his medcial acquaintances in Liverpool, to inquire into the nature of them. Such information he might have easily obtained."

ruptures a sound stomach, must easily have ruptured the tender portion of this.

"Upon the supposition that this hole had been occasioned by some mineral poison, this poison must have acted in one of two ways. It must have destroyed the texture of the stomach; by combining with its substance, and acting upon it as upon dead matter of the same kind; or by exciting inflammation and gangrene. Upon the supposition that the injury was effected according to the first of these ways, by chemical combination, then the quantity of poison required to destroy the texture of a part upwards of six inches in diameter, must have been enormous. The poison, in this case, would have produced almost instantaneous death, and have been found in combination with the destroyed part of the stomach. Why was not the tender part of the stomach submitted to chemical examination, which must, in my opinion, have been decisive of the question, whether the destruction had been occasioned by some corrosive drug, in the way supposed, or not?

"The destruction could not have been occasioned by the poisonous drug exciting inflammation and gangrene; as, in that case, the gangrenous part must either have been separated from the sound, which would have been easily discovered, or a part must have been in a state of high inflammation. But this was not the case. The gentlemen, indeed, admit, that the aperture could not have been occasioned by gangrene. Had this happened, it would not even have inferred the administration of poison, as the stomach is subject to inflammation and gangrene from other causes.

"Had the aperture been occasioned by any acrid substance acting before death, blood vessels would necessarily have been corroded, and would have discharged blood, which would have been ejected by vomiting and stools. Vomiting and purging of blood are too remarkable to have been overlooked, had they occurred.

"The symptoms of the disease, by which this lady was affected, were not those which we know, from the experience of mankind, are produced by the operation of an active poison, especially when administered in such quantity as to destroy any part of the substance of the stomach. Mahon, the elegant and intelligent author of the Medicine Legale, says, that corrosive sublimate, taken in such quantity as to produce death, kills, in a short time, after the most frightful convulsions, and enormous bloody and bilious vomitings and purgings, The derangement of the system arising from the administration of arsenic, is nearly the same with that excited by the use of corrosive sublimate. Excruciating pains in the stomach and bowels, inextinguishable thirst; retching; and the instant rejection of whatever is swallowed; anxiety and intolerable anguish, expressed by moans and lamentations, which no sentiment of precaution could suppress; by restless agitation, and tossing of the body and limbs; hiccough; faintings; convulsions; failure of the voice; inarticulate speech; difficulty in swallowing; and aberration of mind, are among the symptoms which united, or in greater er part, accompany the operation of an active mineral poison, given in a powerful dose.

"Miss Burns, however, does not appear to have suffered any severe degree of pain. She was generally found by the servants lying upon the sofa quietly, and without complaining. She only once complained of pain to any of them, during her illness. From Thursday afternoon, the irritation of the stomach appeared to have been completely removed. From that period she had no vomiting, retchings, nor pain in her bowels. That the strength of the stomach had been in a great degree recovered is proved beyond all doubt, by the quantity of gruel and warm beer which was found in it and in the intestines after death. Such a quantity of nutritive substance could not have been kept by a stomach labouring under the effects of great irritation. The general strength, also, was recruited along with that of the stomach. She could stir more about. So far from being delirious, she appears to have had the most perfect recollection; she continued to give directions respecting the management of the house until the period of her death."

At this place we shall rest, to offer our opinion concerning the various causes which have been assigned for the appearance in the stomach, first bringing before our readers the arguments of the other gentlemen in favour of their opinion, that Miss Burns's stomach was affected by some acrid substance.

1st, It is urged, that it could not be the effect of putrefaction, inasmuch as there were no other symptoms of that process.

2d. That it could not be the effect of disease, as the previous symptoms would not justify such a conclusion.

3d. That it could not have been effected by the gastric juice.

"1st. Because, when the stomach has been found dissolved by the gastric juice, it has been almost only in persons who have died *suddenly* by accident or violence, and in whom the stomach was in the full exercise of its functions, and consequently the gastric juice was in a state of great activity. But Miss Burns did not die suddenly in the sense in which that word is here intended to be used; and she had severe and violent disorder in her stomach before her death; therefore two important conditions were wanting in her case, to render it at all probable that the gastric juice had occasioned the injury in her stomach.

"2dly. Because when the stomach has been found dissolved and perforated by the gastric juice, that fluid has been observed to have acted upon, and dissolved parts of the neighbouring viscera; such as the spleen, and even the diaphragm, and to have penetrated into the cavity of the chest, and acted upon the lungs. But neither the spleen, nor the diaphragm, nor the intestines, nor any other of the viscera, had the slightest appearance of having been acted upon by the gastric juice in the case of Miss Burns.

"3dly, Because during the illness of Miss Burns, the large quantity of fluid which she drank, and which was immediately rejected by vomiting, must have carried off the gastric juice as fast as it was was secreted; and the quantity of fluid found in her stomach must have so diluted and weakened the gastric juice remaining, as to render it unequal to dissolve the stomach itself after death.

"4thly, Because the injury was not in the lowest and most depending part of her stomach, but in the anterior part of the large curvature, which would be the superior surface of the stomach, when the body was lain in a supine position, as it always is after death.

"5thly, Because the appearances in her stomach did not correspond with the effects of the gastric juice upon the stomach, as described by Mr. John Hunter, the only writer who has described them. His description is as follows. "By comparing the inner surface of the great end of the stomach, with any other part of the inner surface, the difference will be obvious. The sound part appears soft, spongy, and granulated, *without distinct blood-vessels*, opake, and thick: the other part" (that is, the part acted upon by the gastric juice) "appears smooth, thin, and more transparent: *the vessels are seen ramifying on its surface, and on squeezing the blood which they contain, from the larger vessels to the smaller, it will pass out at the digested ends of the vessels, and appear like drops upon the inner surface.*" Now, in the injured part of Miss Burns's stomach, which extended more than two inches around the opening, in every direction, there was no trace whatever of any blood-vessel to be found. The structure of this part was completely destroyed.

"Lastly, Because the action of the gastric juice affords no explanation whatever, of the inflammation in the smaller curvature of the stomach, in the *duodenum*, in the small intestines, the liver, or the *fundus uteri*. Nor does it afford any explanation of the sudden and violent illness with which Miss Burns was affected, or of its rapid and fatal termination.

"For these reasons we were of opinion, that the injury in the stomach of Miss Burns, was produced neither by putrefaction, disease, nor the gastric juice. It seemed to us most probable, that her sudden and severe illness, the injury in her stomach, and the inflammation in that organ, and the other viscera above mentioned, were occasioned by one and the same cause; and that such cause must have been some deleterious and poisonous substance, taken into the stomach. Such violent effects must have had an adequate cause; and we know no other way of explaining these effects, but that which we have adopted."

Mr. Carson, on the contrary, deposes, that the pulpy, tender, and ragged appearance described in the report, is the very character of a digested stomach given by Mr. Hunter. In our next we shall show, that whilst these gentlemen seem to contradict each other, they are correct in their quotations; and yet, that Mr. Hunter has not contradicted himself. But it seems as if it would be for ever the fate of that wonderfu man to be misunderstood.

( To be concluded in our next. )

